# 

**Published:** 1859-02

**Authors:** 


Teeth, with full instructions to preserve and keep them beautiful, to the most extreme old age, without the aid of the Dentist; with instructions by which to detect quack dentists, and the propriety of avoiding patronising the same. By H. H. Black, Surgeon Dentist." Price, 25 cents.

We have hurriedly glanced over this production, and find in it much interesting information and many valuable hints and suggestions, which, if properly applied, will go far toward the object aimed at; but we think the Doctor—in giving "instructions to *preserve and keep the teeth beautiful to the most extreme old age*"—claims a little too much—more than can be accomplished in one generation,—while much may be done. There is undoubtedly a large amount of caries induced by constitutional causes, transmitted from parent to child, which will require the coöperative skill of the dentist and physician, with the most vigilant care of parents, for several successive generations; but a beginning in the right direction can not be made too soon, the community can not become too soon nor too well informed on this subject. If it were the loss of the teeth ONLY, it would not be so bad; but comfort, health, and beauty are involved. No person can long enjoy either of those blessings, without good teeth. We hope this little Book will have a large circulation and be the means of doing much good.

### SPECIAL NOTICE.

In an advertisement of MOSES DOLPH, on the cover, of "The Dental Lamp," the language gives a *false impression.*

The last article purchased from him was May 5th, 1857, and prior to that time, the goods were made expressly to my order, at specified prices. My name is, therefore, unnecessarily and *improperly* used, for A PURPOSE!

My arrangements since then are very SUPERIOR. Manufacturing by machinery in large quantities, I am enabled to *sell at* what it *costs* to manufacture by hand, and still make a HANDSOME PROFIT. For particulars, see advertising pages on Catalogues. JOHN T. TOLAND.

### THE CINCINNATI MEDICAL NEWS

Is the title of a monthly paper, edited and published by Prof. A. H. BAKER, M. D., of this city,—which should have been noticed in the November number, but was overlooked. This is a free, bold, and independent publication, and calculated to do much good in the profession, by exposing error and advocating truth. Generally there is too much disposition to "smooth things over." Give us the man who is always ready to "face the music," and such be believe Dr. Baker to be. We bespeak for the *Medical News* a large circulation. Price, fifty cents a year.

### SHERWOOD'S FORCEPS.

For three years (viz: from January 1st, 1856), Mr. H. R. Sherwood has manufactured *exclusively* for me—all his work passing through my hands. At that date, there were about two hundred pairs of Sherwood's Forceps in market. Since that date, I have sold nearly $12,000 worth of his instruments, and can not get them made fast enough to supply the demand. Still I see advertisements of "Genuine Sherwood's Forceps," which I must acknowledge my inability to account for, unless it is the old shapes and styles made several years ago. Of these we have daily offers to trade or exchange, which we generally decline, because we want only the best of every thing; and in Sherwood's Forceps, such improvements have been made, that the *old* styles would not be *recognized* in company with the *new*, except by the *name*. John T. Toland.

### THE DENTAL ENTERPRISE.

A monthly publication, bearing the above name, has fallen under our notice. It is an enterprise of rare merit; and we would advise all to subscribe. It is published by Henry Snowden. Price, fifty cents a year.

☞ "*The Cincinnati Dental Lamp*"—Is the title of a new aspirant to *fame* and *wealth* in dental literature. We have not been favored by the publisher with a copy, but by *accident* obtained the November issue, Vol. I, No. 1.

From the prospectus we learn, that "the *Lamp* will be published quarterly and will be what its name imports," "*a light to dispel the darkness which hangs brooding over Dental Science.*" Truly, we trust the *Lamp* will be able to accomplish the *task* which it has assumed. The *Lamp* is neatly printed, on good paper, and filled with matter highly interesting to the profession. The intelligence, sprightly wit and humor of the Editor, Dr. J. M. Brown, are readily observed on every page.

The *Lamp* will be furnished to subscribers, for twenty cents a year. Verily this is *cheap enough*, those who can not afford to subscribe for the *Lamp*, deserve to remain in *darkness*.

We wish the *Lamp* success on its mission; but if the Editor expects a "liberal profession" to reward him for his labors, before his expectations are realized we fear he will have occasion to say with the poet—

"The Lamp must be *replenished*, but even then,
It will not burn so long as I must watch."
But while the *Lamp* contrives to burn,
The vilest sinner may return.

---

☞ *The Peninsular and Independent Medical Journal*—Devoted to Medicine, Surgery, and Pharmacy.

We are in receipt of several numbers of this valuable Journal. It is ably and independently conducted, by a highly scientific corps of Editors, and is calculated to effect much good by its independent tone and the large amount of practical and scientific information with which each number is laden. We bespeak for it a large circulation.

The *Journal* contains 64 pages exclusive of advertisements, and is issued monthly, at Two Dollars a Year in advance. Published by Higby & Stearns, 162 Jefferson Avenue, Detroit, Michigan.

---

☞ *The Lewiston Falls Journal* (Lewiston, Maine)—Edited by Nelson Dougley, jr.; is a valuable family newspaper, which deserves a large circulation and close perusal. We notice in it an article on "Popular Dental Knowledge," which we commend to attention of all readers of the *Journal*. This is a step in the right direction. Let the *people* be *educated* in the structure, value, *use* and *abuse* of their teeth, and Dental Science will rise to the dignity and importance to which it is entitled. For this result, we must depend on the medical profession and the secular Press.

---

### APOLOGY.

*Indiana State Dental Association.*—We had the pleasure of being present on the 28th and 29th of December, at a meeting of the above named Society, in the city of Indianapolis, and were well paid for the loss of time and expense; indeed, it has rarely been our fortune to witness so much systematic business order, intelligence and harmony of feeling, in any gathering of this kind. Each man seemed to have come for *a purpose*, which he fully understood, and was earnest in executing.

The discussions were interesting and ably conducted,—the details of which we *expected* to publish in this number; but through some mishap of our friend "Perine," (which he can explain for himself), we have neither notes, mem. or report. We *loaned* our "*notes*" to Dr. P., to make up a report for the New York *Journal*, under promise of furnishing us with advance sheets by the 10th of January, at latest. We have waited from day to day, until the 10th of February, but no report cometh; we can wait *no longer;* as we have neither our own notes nor Dr. Perine's report, we must go to press without it, with the promise that the report will be found in the March number of the *Dental Register of the West*,—together with a report of the College Commencement, College Association, and Proceedings of the MISSISSIPPI VALLEY ASSOCIATION, all of which take place February 20th, 21st, 22d, 23d and 24th. The meetings of this Society are attended with much interest, and will well repay a visit from the profession of the neighboring States.

If the friends of the Indiana Association will forgive us this time, we will promise the like shall not occur again, as we have resolved to adopt a new principle in reporting, viz: "Neither borrow nor lend."

### THE DENTAL REPORTER.

This closes the *first* volume of the *Dental Reporter*, as a subscription Journal.

When we commenced its publication, our aim was to be the *cheapest* in the world, and furnish fresh and reliable information on all subjects interesting to the profession; to which we expected a hearty and enthusiastic response, in showers of 25 cent pieces We thought every dentist in the United States, besides many physicians and others, would become immediate subscribers, so as to make a list of at least 10,000. Well, probably there are that many who would like to consider themselves subscribers to the "Reporter," but they forget this is a fast age—we can't wait, they have been too *slow*. Our list has not exceeded 4,000, which, at 25 cents, won't pay *first cost!* while others have started at 20 cents, 10 cents, and even 5 cent.

The *Reporter* has been devoted to the profession, not as an "advertising journal," for its pages have been kept free from advertising, except in a separate department, such as every literary journal, has annexed for advertising purposes.

If we can not be the cheapest and have the largest circulation, we are determined to be among the most respectable, and therefore concluded to take ourself out of *bad company*.

We had resolved to raise the price of next volume, to $1 a year, and have already received several subscriptions for the next volune; but within a few days, we have concluded to purchase tho DENTAL REGISTER OF THE WEST, with its books, credits, and subscription list, and will hereafter be proprietor and publisher of the same, and will therefore abandon the *Reporter*, which will end its existence with its first volume. Those who have paid on the second volume of *Reporter*, can have the amount applied on the *Register*, or the money refunded, as they choose, by notifying us of their desires.

Drs. J. TAFT and GEO. WATT will continue as Editors of the *Register*,—the subscription price is $3 a year, in advance.

After the close of the present volume which will end with the June number, the *Dental Register* will be issued MONTHLY, instead of Quarterly,—and at the same price per year.

### DENTAL REGISTER.

Having become proprietor and publisher of this highly respectable and popular Journal, I will cease the publication of the *Denial Reporter*, and devote myself to the interests of the the *Register*. Drs. TAFT and WATT will continue as Editors.

At the expiration of the present volume, which ends with the June number, the *Dental Register* will thereafter be issued monthly, at the *same* price as heretofore, viz: $3 a year, in advance, only that *advance* payments will be insisted upon. In changing from a quarterly to a monthly, both *labor* and *expense* will be trebled; but as the advantages to the profession are so apparent, I anticipate a like increase in the subscription list. All friends of the movement will at once see the propriety of exerting themselves to increase the circulation of such a Journal. Its communications, reports and selections will always be fresh and new—always in advance of every other journal. It will be issued and sent promptly, to all prompt paying subscribers.

New cash subscribers respectfully solicited.

Those *indebted* to the "REGISTER," will confer a favor by prompt remittance of balance due, as I wish to square up the old books, and start anew. JNO. T. TOLAND.

FRANCIS' GALVANIC AND ELECTRO-MAGNETIC PROCESS FOR EXTRACTING TEETH WITHOUT PAIN.

Office Rights, for the full term of the Patent, including complete apparatus, with instructions, will be sold for $60.

Those who have a Battery, will be furnished with a Right for $50. JNO. T. TOLAND,

38 *West Fourth Street, Cincinnati, O.,*

*Agent for the West and South.*

### GALVANISM IN EXTRACTING TEETH.

At the stated meeting of "Pennsylvania Association of Dental Surgeons," held December 7th, 1858, the Committee on Galvanism made the following report, which, on motion, was adopted:

MR. PRESIDENT: The committee appointed by the Association to investigate the merits of applying the galvanic current in the extraction of teeth, would beg leave to report, that they have attended to the duty assigned them.

Immediately after their appointment, the members of the committee were furnished with galvanic batteries, and instructed how the current should be employed, by the agent of the patentee. These bateries have been used, and the instructions followed by the committee in their investigations. Desirous of arriving at an impartial and unbiased opinion, and believing that this could only be obtained from a somewhat lengthened series of experiments, the committee have permitted several months to elapse before presenting their report.

Their experience, which has been gained in *private practice*, has varied from time to time. At one period, certain members were disposed to believe that they recognized in the application of the galvanic current, all that had been claimed for it, and all that had been said in its favor; but a later experience and more careful observation convinced them to the contrary. They have found that although in a certain proportion of cases they have had evident alleviation of suffering, and in some the assurance that the operation has been absolutely painless, yet that in the majority of instances, the pain of extraction has been unmitigated, and in some cases the application has been denounced as productive of the most excruciating suffering.

With the view of ascertaining how far galvanism was instrumental in alleviating suffering, the operator, when engaged in extracting a number of teeth for one individual, would occasion-

### Error in the Dental Register of the West.

Dr. Osmond requests us to state that, owing to the blunder of the individual who reported the proceedings of the Mississippi Valley Association, he was made to say, in the course of a debate on the causes of decay of the dental organs, that "In most American constitutions, *muriatic acid* is produced in too great a quantity to be consumed by the lungs." He trusts that those who are acquainted with him consider that he is sufficiently "*posted*" in Physiology, to be incapable of stating such a gross error. The report should read, "In most American constitutions, *carbon is taken* in too great a quantity to be consumed by the lungs." As to the *muriatic acid*, it is the acid of the gastric juice, according to our best Physiological authorities; combined with pepsin, it acts upon the nitrogenized substances, and also acts as a solvent of the phosphate of lime, consequently, when it is neutralized by alkalies, it loses this latter power; hence a deficiency of that salt, and a tendency to caries. This was discussed at length, but not reported.

Taken altogether, the report was not only meagre, but partial. Ideas advanced by younger members of the profession, in accordance with the recent discoveries in physiology, were either meagerly reported or omitted, to make room for the *lead* of one of the "great guns," which has been so often re-cast and shot over again, that it now contains considerable *dross*.

### Exclusive Agencies.

We do not wish to call in question the veracity of any one, but when we see, paraded as "*exclusive agencies*," Goods which are sold by *every dealer*, and some of which are not put on agency to any person, there must be either a very slight regard for truth, or a great amount of *ignorance*.

But suppose it were *true*, what would be the advantage? Is any one fool enough to suppose, in this day of progress, that any desirable article of merchandise can be kept in the hands of a few individuals? An *agent* is always under a disadvantage, from the fact that he is under the direct control and discretion of the *proprietors*, and can act only as they *dictate*, and must always *pay more* for his goods.

No manufacturer can or will put goods out on *commission* as low as he will SELL the same articles—1st. Because he has all the risk, and 2d. Because he has to lay out of the money a long time, with great uncertainty as to amount sold. For example: Patent medicine which are commissioned at $8 per dozen, are *sold* at $7, and some much lower. There is always a difference of from 12 to 25 per cent.

### A NEW QUARTERLY.

THE

### DENTAL REPORTER,

### AN INDEPENDENT JOURNAL,

DEVOTED TO

### Dental Progress, and Improvement in the Manufacture and Use of Instruments and Materials.

**EDITED BY JOHN T. TOLAND, 38 WEST FOURTH STREET,**

**CINCINNATI, O.**

### TERMS.

| | |
|---|---|
| One copy one year,........................................................ | 25 cents. |
| Five copies, one year to one address,............................. | $1 00 |
| Thirty copies, one year to one address,........................... | 5 00 |
| One hundred copies, one year to one address,................... | 15 00 |

Those who will get up clubs, can thus realize a handsomo profit for themselves.

*All Subscriptions must be Cash in Advance*, as at this low price we can not afford to keep *book accounts*, nor incur the expense of sending out *collectors*. Every Dentist, Physician and Druggist should have it.

### TO ADVERTISERS,

The "REPORTER" offers superior advantages to any other Dental Journal, its circulation being much larger than any other, and from three to five times as great as some. The first No., 3,000, the present number 4,000 copies, and expect to increase with each issue.

The circulation extends to *every state* in the Union, but more especially *west* of the Allegheny mountains, where it goes to *every town* of any importance. It goes into the hands of the most INTELLIGENT and LIBERAL portion of the community, viz: *dentists*, *physicians* and *druggists*. Being a *book of reference*—it remains on the table in the offices of subscribers, where it is seen and frequently examined by the numerous visitors, who are waiting for operations or advice.

### TERMS OF ADVERTISING.

| | |
|---|---|
| One page for one year,................................................................ | $40 00 |
| Half a page, " ........................................................ | 25 00 |
| One fourth of a page, one year,................................................. | 15 00 |
| One eighth page, one year,.......................................................... | 10 00 |

*Special* advertisements by *contract*. Advertisements to be *paid in advance*.

Those who wish to avail themselves of the facilities of *extensive* advertising at *small expense* as above, will please forward their copy, accompanied by a *remittance* to *pay* for the amount of *space* they wish to occupy, according to the above terms, this will save the time and trouble of correspondence, and insure *prompt insertion*.

